# Analysis of Fat Content with Special Emphasis on Trans Isomers in Frequently Consumed Food Products in Egypt: The First Steps in the Trans Fatty Acid Elimination Roadmap

**DOI:** 10.3390/nu13093087

**Published:** 2021-09-02

**Authors:** Ghada Ismail, Randa Abo El Naga, Maysaa El Sayed Zaki, Jana Jabbour, Ayoub Al-Jawaldeh

**Affiliations:** 1 Clinical Pathology Department, Faculty of Medicine, Ain Shams University, Cairo 11566, Egypt; ipcuh@scu.eg; 2 Noncommunicable Diseases and Mental Health, World Health Organization, Egypt Country Office, Cairo 11516, Egypt; abouelnagar@who.int; 3 Department of Clinical Pathology, Faculty of Medicine, Mansoura University, Cairo 35516, Egypt; maysaazaki5@hotmail.com; 4 Nutrition Department, School of Health Sciences, Modern University of Business and Sciences, Beirut 113-7501, Lebanon; 5 Regional Office for the Eastern Mediterranean (EMRO), World Health Organization (WHO), Cairo 11371, Egypt; aljawaldeha@who.int

**Keywords:** trans fatty acids, saturated fatty acids, fat profiling, Egypt

## Abstract

Trans Fatty Acid (TFA) intake is a risk factor for coronary heart diseases and cancer. Egypt, considered among the highest TFA consumers in the world, lacks proper dietary analysis of TFAs. This cross-sectional study aimed to analyze TFAs in traditional and frequently consumed food products. A market survey was conducted to identify products and brands that are mostly consumed in major governorates in Egypt. Laboratory analysis allowed for the profiling of TFAs, and saturated and unsaturated fatty acids. Products having more than 2 g of TFA/100 g of fat were considered to have an elevated TFA content. Commonly consumed food items (*n* = 208) in the Egyptian market were identified. On average, 34% of the products exceeded the TFA limit. Sambosk meat, a traditional meat item, had the highest TFA content of 5.2%, followed by foods fried with used oils. Oriental sweets had a TFA content three times higher than that of doughnuts. The fast-food group had the largest proportion of TFA-rich products, followed by the canned and frozen item groups and confectionaries. This study revealed that around one third of products in the Egyptian market have a high TFA content. This calls for urgent legislative action to regulate composition.

## 1. Introduction

Compared to unsaturated fatty acids, Saturated Fatty Acids (SFAs) are associated with increased serum levels of low-density lipoprotein, odds of Cardiovascular Diseases (CVDs) and incidence of cancer [1,2]. Several regulatory agencies recommended limiting SFA intake to 10% or less of daily caloric intake [3,4,5]. Industrial Trans Fatty Acids (TFAs) were introduced in the 20th century to lengthen products’ shelf lives, reduce costs, and produce solid forms of vegetable oils. TFAs, originally considered a healthy substitute for SFAs [6], were later recognized as more harmful nutrients, associated with an increased risk of CVDs and mortality [7,8] and requiring dietary limits through voluntary and legal reforms [9]. The World Health Organization (WHO) set guidelines to limit TFA intake to 1% of energy intake and identified the elimination of TFA from the global food supply as a health priority in its 2019–2023 agenda [10]. To meet this aim, the WHO and Resolve to Save Lives introduced, in 2018, the REPLACE roadmap, a comprehensive scheme to eliminate industrial TFAs. This roadmap recommended an analysis of TFAs in food sources, the substitution of TFAs with unsaturated fats, and the establishment of legislative actions to limit TFA food composition [11]. Best-practice TFA policies have been implemented in 14 countries, covering only 8% of the world’s population, where they have been efficient in limiting TFA dietary content and intake [10,12]. These best-practice mandatory approaches have been mainly applied in high-income countries [10].

Egypt, a low-income country in the Eastern Mediterranean Region (EMR), has been considered among the highest TFA consumers in the world [13]. Egypt has lacked proper dietary analysis and monitoring of SFAs and TFAs. A majority of accredited food laboratories release reports of fatty acid profiles without mentioning TFAs, and nutrition labels do not incorporate fat profiling. Moreover, Egyptian food composition tables date back to 1996 and 2006, they have not been frequently updated, and they lack data on fatty acid types [14]. They were published during the period when palm oil started to be promoted as a healthy and cheap plant oil and margarine was used in subsidized food items. Fortunately, Egypt recently made a national commitment to eliminate TFAs from its products [10]. This cross-sectional study addressed the first step of TFA elimination: the fat profiling of commonly consumed food items and their comparison to international standards [15].

## 2. Materials and Methods

This cross-sectional study was conducted in February and March 2019 and involved two steps: a market survey and a food analysis. The market survey, as outlined below, identified commonly consumed food sources in the Egyptian market that would benefit from a TFA analysis, whereas the dietary assessment phase involved the analysis of food products’ fat profiles and started from the second week of February.

### 2.1. I-Identification of Commonly Consumed Food Items

A literature review of the possible dietary sources of TFA in the Egyptian market was conducted and shared with an expert committee of public health practitioners, nutritionists, food analysts, and representatives from the WHO and the Central Laboratory in Egypt. A market survey of commonly consumed items was conducted in the following governorates in Egypt: Al Qalyubiya, Cairo, and Giza. Surveys were conducted through convenience sampling in different urban and rural areas representing several socioeconomic levels. The identification of products commonly consumed by residents was completed through field visits to groceries and bakeries in rural regions. An assessment of products that were most sold in urban areas was performed in 3 hypermarkets, selected based on their popularity. The evaluation of products mostly sold from oriental carts (*n* = 10) was conducted for sweets (Zalabia, Basbosa, and others) and salty items (falafel (deeply fried patted beans), fried potatoes and eggplants, etc.). The identification of meals commonly eaten out was performed in restaurants (*n* = 12) serving Western fast food (Western fast-food chains as well as local restaurants serving Western fast foods) and those serving traditional Egyptian food across the two governorates.

### 2.2. II-Analysis of the Fatty Acid Profile

Three samples of 100 g were collected for each brand/item and were delivered to the Central Laboratory of the Ministry of Health in Egypt for laboratory analysis. To determine their complete fatty acid profiles, selected samples were analyzed as per the WHO protocol, outlined below [16].

(a) Each sample was prepared by thoroughly mixing equal weights or volumes of three samples from different batches of the same food item. This mixing method ensured the homogeneity of each sample.

(b) Fats were extracted from food items through hydrolysis (acidic hydrolysis for most food items or acidic and alkaline for products containing industrial and ruminant TFAs). Fatty acid profiling was conducted using the Gas Chromatography (GC)–Flame Ionization Detector (FID) method. After mixing the extracted fat with triheneicosanoin, the mixture was converted to Fatty Acid Methyl Esters (FAMEs) through methylation. The FAMEs were then separated through GC and then identified using relevant standards (SUPELCO 37 Component FAME MIX. CRM47885, Sigma-Aldrich, Saint Louis, MO, USA). Analysis revealed the grams of fatty acid per 100 g of the food sample and the content as the % of the total fatty acids. Outlined below is a description of the instrument and oven program used, as well as the adopted Mass Spectrometry (MS) methodology.

- Instrument used: GC/Mass Selective Detector 5977A, Agilent, Palo Alto, CA, USA.
- Column used: Agilent, DB 225 ms 60 m × 250 μm × 0.25 μm.
- Oven program: 40 °C for 1 min and then 7.2 °C/min to 195 °C, and 2.3 °C/min to 230 °C for 15 min.
- Inlet: Split mode; split ratio: 50:1; Liner Agilent 5190-2294: 990 μL; Inlet Temperature: 250 °C; Auxiliary Temperature: 250 °C.
- MS acquisition scan parameters: Low Mass: 45; High Mass: 550.00.

### 2.3. Ethical and Statistical Considerations

This study did not require ethical approval from an ethics committee, as it did not involve the collection of identifiers from human subjects. Descriptive data are presented in the study as counts and percentages. The limit for TFA content in 100 g of fat was 2. Items exceeding the 2% limit were considered to have an elevated TFA composition [15].

## 3. Results

**Figure 1:**
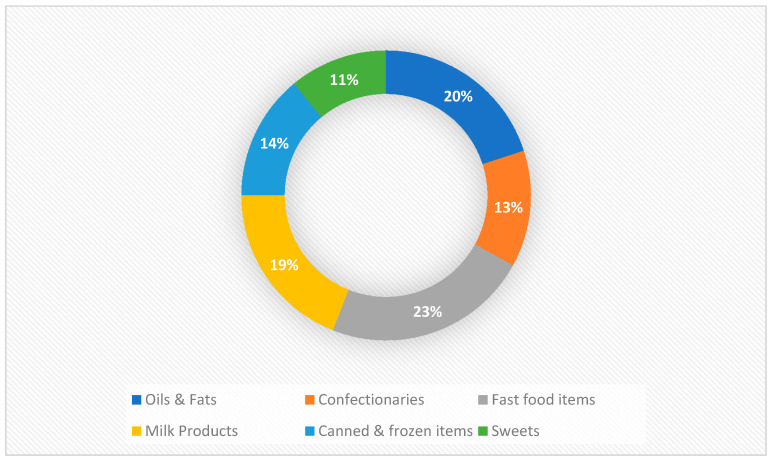
Products analyzed by food categories.

A total of 208 brands of commonly consumed food items in the Egyptian market were identified in the following categories: fats and oils (*n* = 42), milk and milk products (*n* = 40), confectioneries (*n* = 27), canned and frozen items (*n* = 29), fast-food items (*n* = 48) and sweets (*n* = 22) (Figure 1).

In the fats and oils group, mixed oils had the highest content of TFA, followed by sunflower oil and mayonnaise (2.9%, 2.6%, and 2% of the total fat content, respectively). The lowest TFA level in this food category was found in sesame paste (Tehina). It is worth mentioning that nearly 50% of the fat in margarine and yellow butter was SFA, and one brand of olive oil revealed a TFA content of 0.4% of total fats (Table 1). An analysis of the milk products revealed that all the products had an elevated SFA:MUFA:PUFA ratio, with processed cheese having the highest ratio of 5.5:3.1:1.0. The highest content of TFA in a milk product was found in Roomy cheese (2.2% of fat), followed by processed cheese cubes (1.8% of fat) (Table 1).

The measurement of TFA levels in 27 brands of biscuits, cakes, crackers, and potato chips revealed that chocolate cakes and biscuits had the highest contents of TFA (2.6% and 2.0%, respectively) followed by potato chips (1.5%). Items in this food group had a rich SFA content, with plain biscuits having the highest ratio across all the items, with a ratio of SFA:MUFA:PUFA of 7.0:0.3:1.0 (Table 2). Frozen traditional and Western items such as frozen Kafta, Kebba and pizza had a TFA/fat content that exceeded 2.2%. In this food category, chicken cubes and frozen Mombar (sheep intestines stuffed with rice) had the highest SFA:MUFA:PUFA ratios (Table 2).

**Figure 2:**
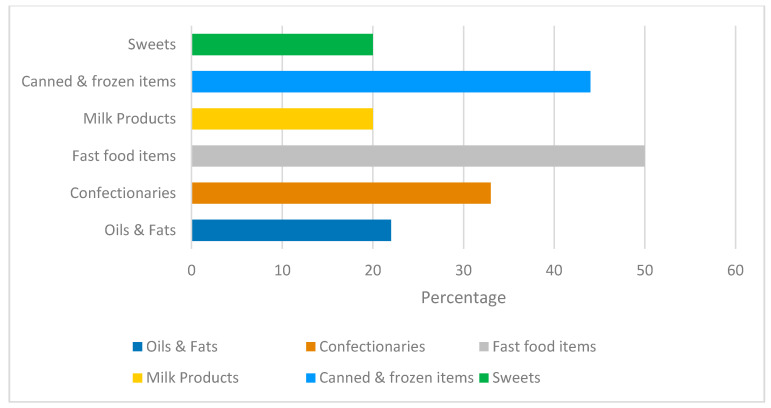
Percentage of products exceeding TFA limits by food category. TFA limit: trans fatty acid content > 2 gm/100 g fat.

Fast-food items had a high TFA content, with some traditional dishes such as Sambosk meat (deeply fried pastries with meat) having 5.4% TFA of fat content, followed by falafel and kabbab (mixed meat grills). It is worth noting that the frying oil had a major impact on the TFA content, with falafel and potatoes made with used oils having more than four times the content of those prepared with new oil (Table 3). In the sweets’ category, oriental sweets had the highest TFA content, three times higher than doughnuts and Halawa (a traditional sweet made with sesame paste). Doughnuts had an elevated SFA:MUFA:PUFA ratio, similar to that of oriental sweets (Table 3). On average, 34% of the analyzed products exceeded the TFA limit (data not shown). Figure 2 illustrates the percentage of products exceeding the TFA limits (2 g/100 g of fat) presented by food categories. With 50% of the analyzed products exceeding the TFA limit, the fast-food group had the largest proportion of non-compliant products, followed by the canned and frozen item groups and confectionaries (Figure 2).

## 4. Discussion

In a country leading in TFA consumption, profiling food products’ fat content is of vital public health importance. The fat profiling of products available in the Egyptian food markets revealed that all the food groups had an elevated TFA content, with fast foods, canned and frozen items, and confectioneries in the lead. Some traditional Egyptian fast-food items and sweets had TFA contents that surpassed their Western counterparts. The saturated fat content was elevated in biscuits, fried eggplants, luncheon beef, and some dairy products such as milk and cheeses.

In our analysis, oil used five times or more had four times the TFA content of unused oil. Prolonged heating, repeated use of oils for frying, and adding new oils to old ones can all explain this elevated TFA content. Moreover, used oils may also have an elevated TFA content, as they have remnants of fatty items heated in them such as meats, poultry, etc. These findings are in line with the literature that reveals a negative impact of the repeated use of oil, especially for frying [17]. Of interest, one olive oil mixture had a borderline elevated TFA content. This may be due to the method of oil extraction conducted at a high temperature and may be associated with mixing the olive oil with cheaper types of oils. This may also be explained by the elevated temperature oils were exposed to during processing, the fact that hydrogenated oils may have been added during preparation to extend their shelf lives, and the physical refining they underwent [18].

Around 33% of confectionaries had an elevated TFA content, and most of them had an elevated SFA composition. These findings are similar to those observed in Polish evaluations of confectionaries and pastries [19,20]. Moreover, some traditional Egyptian meat items such as frozen keba, kafta, mombar, and Sambousk meat had TFA contents exceeding those of Western items such as nuggets and luncheon beef and similar to frozen pizza. The same observation was noted with oriental sweets, where the content surpassed that of doughnuts. Western dietary patterns have been associated with increased risks of NCDs among youth and adults compared to Mediterranean dietary patterns [21,22,23,24,25]. However, this study highlights that some traditional items consumed in the diets of the Mediterranean region may be more harmful than Western food items and do not fit in the Mediterranean dietary pattern. These findings are in line with analysis of traditional food items in Lebanon, a neighboring Mediterranean country, that revealed that some traditional items are energy dense and have an elevated content of carbohydrates and/or sodium [26,27].

The Egyptian population faces several nutritional challenges. Egypt is among the top 20 countries worldwide for the number of children with chronic malnutrition and stunting [28,29]. Obesity rates are elevated and on the rise among children, youth, women of reproductive age, and all adults [30,31]. NCDs contribute to around 85% of all the deaths in the country, surpassing the worldwide average of 70% [32]. Our analysis showed that TFAs were found to be elevated in around 34% of the food groups on average. Given the harmful effects of TFA on NCDs, this study helps to explain the elevated rates of NCDs in the country and identifies the urgent need for product reformulation and for progress in the WHO REPLACE roadmap [11].

In the 1990s, Canada was leading worldwide in TFA consumption, with around 4% of caloric intake originating from TFAs. After implementing legal reforms and collaborating with stakeholders from the food industry, a significant reduction in TFA content was observed, with more than 95% of assessed products found to be compliant with TFA limits in 2014 [33]. Even though some manufacturers replaced TFAs with SFAs, the majority increased unsaturated fat composition rather than SFAs [34]. More recently and in a country closer to Egypt, Saudi Arabia established the Healthy Food Strategy that incorporated legal reforms to reduce TFAs [35]. In 2020, it became the first country in the EMR to have a ‘best practice TFA policy’ that eliminated industrially produced TFAs [10]. The success of Canada and Saudi Arabia in reducing TFA content was grounded in the collaboration with key stakeholders in the food industry, the development of several affordable mechanisms for food establishments to conduct TFA analysis, and the empowerment provided by governmental entities to consumers such as the inclusion of TFA on nutrition labels [34,35]. The implementation of ‘best practice TFA policies’ has mainly occurred in high-income countries [10]. However, additional challenges are present in developing countries such as Egypt. These include resistance towards regulations by the food industry, a lack of governmental interest in such reforms, and the elevated cost of healthier alternatives for the manufacturers and consumers [36,37]. In Egypt, around 30% of the population live below the poverty line, and the illiteracy rate exceeds 25% [38,39]. Low-income customers may be more sensitive to price changes and may be more likely to consume products with higher TFAs if they cost less. In addition to socioeconomic status, education plays a role. In countries requiring the labeling of TFA contents in foods, consumers with lower literacy rates are less likely to use nutrition labels and thus may continue to purchase products with high amounts of TFAs. The adoption of consumer-friendly labels, such as the Front of Pack Nutrient Label (FoPNL), has been evolving in the EMR region through voluntary and mandatory schemes [40]. Considerations for FoPNL implementation in Egypt should be made to empower consumers to make healthy decisions.

This study has several strengths and limitations. On the one hand, the limitations included the use of convenience sampling when choosing food outlets. Moreover, the sampling relied on data from major but not all governorates in Egypt. On the other hand, this study has several strengths. The methodology of fat profiling analysis was robust and followed the WHO recommendations. Moreover, even though data were derived from two governorates only, they are considered to be representative of the country given their size and that citizens residing there come from all of Egypt. Moreover, food products were selected from diverse food outlets serving traditional and Western cuisines in urban and rural areas and from various socioeconomic levels. The selected food products are likely to represent commonly consumed items across the country.

## 5. Conclusions

With the review of the TFA contents of food products, the first step of the TFA elimination roadmap was addressed in Egypt. A large proportion of products were found to be rich in SFAs and TFAs, which may explain the heavy burden of NCDs in the country. Next in line, the Egyptian government can validate these findings across the country and should collaborate with the food and beverage industry and with food outlets to reformulate their products. The enforcement of relevant legal reforms and the establishment of systems to monitor adherence are of vital importance for the success of this initiative. To complete the puzzle, consumers will need to be empowered through educational interventions and the adoption of consumer-friendly nutrient labels that facilitate healthy decision making.

## Figures and Tables

**Table 1:** Fat profiles of fats, oils, and milk products.

Food Item	Brands, *n*	Fat %	TFA g/100 g Fat	TFA/100 g Food	SFA:MUFA:PUFA
**Fats and oils**					
Sunflower oil	6	100	2.6	2.6	1.1:1.8:0.41
Olive oil	3	100	0.4	0.4	1.0:3.5:1.0
Corn oil	3	100	0.2	0.2	1.0:2.0:4.0
Mixed oil	8	100	2.9	2.9	1.2:2.0:1.0
Butter (yellow)	3	81	1.2	0.3	1.4:1.0:0.2
Margarine	10	72	0.9	0.2	1.3:1.0:0.2
Coconut margarine	1	73	0.8	0.6	1.3:1.1:0.2
Mayonnaise	4	75	2.0	1.5	1.0:1.7:2.8
Tehina	4	54	0.8	0.05	1.0:2.0:2.6
**Milk Products**					
Liquid milk (full cream)	5	17	2.1	0.3	2.5:1.0:1.0
Powder milk (full cream)	4	3	1.4	0.04	4.99:1.0:0.3
Powder milk (skimmed)	4	0.5	1.0	0.005	3.1:2.8:1.0
Coffee creamer	3	36	1.0	0.4	1.34:0.1:0.02
Roomy cheese	4	30	2.2	0.7	2.0:1.0:0.06
Mozzarella	4	10	0.9	0.09	3.3:4.5:1.0
Feta cheese	4	21	1.5	0.4	5.5:4.2:1.0
Creamy spread	4	30	1.7	0.5	2.34:1.21:0.7
Processed cheese with cream cubes	3	37	1.8	0.7	2.3:1.0:0.7
Processed cheese triangles	5	25	1.7	0.4	5.5:3.1:1.3

SFA:MUFA:PUFA reflects the ratio of saturated fatty acids to monounsaturated fatty acids to polyunsaturated fatty acids.

**Table 2:** Fat profiles of confectionaries, and frozen and canned products.

Food Item	Brands, *n*	Fat %	TFA g/100 g Fat	TFA/100 g Food	SFA:MUFA:PUFA
**Confectionaries**					
Plain biscuits	5	17	0.2	0.03	7.0:0.3:1.0
Biscuits with chocolate	4	39	2.0	0.8	4.0:3.0:1.0
Plain cake	4	9.8	0.5	0.003	2.0:1.0:0.8
Chocolate cake	4	23	2.6	0.6	2.6:1.0:0.9
Crackers	5	20	1.3	0.3	1.0:1.0:0.3
Potato chips	5	38	1.5	0.6	1.2:1.0:0.3
**Frozen and canned items**					
Frozen Pizza	3	15	2.2	0.3	2.11:1.0:0.2
Frozen keba (meat and burghul dish)	3	10	2.2	0.2	0.74:1.0:2.3
Frozen kafta (meat dish similar to kebab)	3	14	3.2	0.5	1.0:2.0:0.2
Frozen mombar (sheep intestines stuffed with rice)	3	9.8	2.1	0.2	3.2:0.5:1.0
Frozen nuggets	3	23	1.3	0.3	2.0:1.0:0.8
Luncheon (beef)	4	14	1.5	0.2	4.1:4.6:1.0
Canned luncheon (beef)	4	14	0.4	0.05	1.3:1.8:1.0
Canned tuna	4	24	0.2	0.05	1.0:1.6:4.0
Chicken cubes	2	12	0.7	0.08	3.7:1.0:0.2

SFA:MUFA:PUFA reflects the ratio of saturated fatty acids to monounsaturated fatty acids to polyunsaturated fatty acids.

**Table 3:** Fat profile of fast-food items and sweets.

Food Item	Brands, *n*	Fat %	TFA g/100 g Fat	TFA/100 g Food	SFA: MUFA: PUFA
**Fast-food items**					
Hamburger A *	2	8.50	1.3	0.1	1.0:1.1:0.3
Hamburger B *	2	9	1.9	0.2	1.0:1.0:0.3
Hamburger C *	2	11	1.5	0.2	1.0:1.0:0.1
Shawarma	3	9	2.1	0.2	1.0:1.1:0.1
Fried chicken	3	17	2.7	0.5	2.6:1.5:1.0
Falafel—new oil ¥	3	17.8	0.9	0.2	1.0:0.9:0.3
Falafel—in used oil ¥	5	17.8	4.8	0.9	2.0:1.0:0.3
Fried eggplants	3	23	2.1	0.5	7.0:6.0:1.0
Fried potatoes—new oil ¥	4	44	0.5	0.2	1.0:1.3:2.0
Fried potatoes—used oil ¥	3	44	4.7	2.1	1.04:1.1:0.3
Sambosk cheese (pastries with cheese)	4	25	1.8	0.5	1.11:1.1:0.7
Sambosk meat (pastries with meat)	4	30	5.4	1.6	1.12:1.0:0.5
Koshary (rice, lentils, pasta, and onions)	4	5	0	Zero	1.0:2.6:4.8
Kabab (mix grill a) **	2	20	2.4	0.5	1.0:1.1:0.1
Kabab (mix grill b) **	2	21	2.9	0.6	1.3:1.0:0.09
Kabab (mix grill c) **	2	25	3.6	0.9	1.0:1.0:0.4
**Sweets**					
Oriental sweets from specialized sweet shops	5	9	1.7	0.2	1.6:1.1:0.3
Oriental sweets from bread bakeries	5	12	3	0.4	1.9:1.0:0.2
Zalabia—new oil (deeply fried flour batter)	4	30	0.1	0.03	1.03:7.0:12
Doughnuts	4	25	0.9	0.2	2.0:1.3:1.0
Halawa	4	44	0.7	0.3	1.0:1.2:1.4

SFA:MUFA:PUFA reflects the ratio of saturated fatty acids to monounsaturated fatty acids to polyunsaturated fatty acids. * Hamburgers A, B, and C categorized according to the socioeconomic levels of their sources and their target customers. Category A refers to the lowest socioeconomic level, and C, to the highest. ** Mix grills a, b, and c are categorized according to the socioeconomic levels of their sources and their target customers. Category A refers to the lowest socioeconomic level, and C, to the highest. ¥ New oil:oil used for frying fewer than 5 times; used oil:oil used for frying 5 times or more.

## Data Availability

Some 3rd Party Data Restrictions apply to the availability of these data. Data were obtained from the Reference Laboratory for Egyptian University Hospitals and are available from the author Ghada Ismail with the permission of the Reference Laboratory for Egyptian University Hospitals.
